# 537. Clinical Application of Circular Pneumatic Dermatomes in Burn Wound Management

**DOI:** 10.1093/jbcr/irag033.234

**Published:** 2026-04-06

**Authors:** Carson Bair, Armen Brotgandel, Emily Coughlin, Ryan Hoffman, Jared Troy, Sai Ram Velamuri

**Affiliations:** Tampa General Hospital Regional Burn Center, Allentown, PA; University of South Florida Health Morsani College of Medicine, Tampa, FL; University of South Florida Health Morsani College of Medicine, Tampa, FL; University of South Florida Health Morsani College of Medicine, Department of Plastic Surgery, Tampa, FL; University of South Florida Health Morsani College of Medicine, Department of Plastic Surgery, Tampa, FL; University of South Florida Health Morsani College of Medicine, Tampa, FL

## Abstract

**Introduction:**

Excisional management of burn wounds is a cornerstone of surgical care. Traditional sharp excision allows precise removal of devitalized tissue but may be limited by surgeon fatigue. A circular pneumatic dermatome is designed to enhance efficiency in wound excision and graft harvest. This retrospective cohort study compared operative times and transfusion rates for burn wound excision using a circular pneumatic dermatome.

**Methods:**

All patients undergoing burn wound excision between January 2023 and June 2024 at a verified burn center were included. The excision tool, total surface area excised, and number of Lund and Browder–defined anatomical sites excised were recorded. Primary outcomes were operative time and intraoperative transfusion requirements. Outcomes were compared using Mann–Whitney U and Fisher’s Exact Tests, with *p*<.05 considered statistically significant.

**Results:**

A total of 136 patients underwent 210 excision procedures. The circular pneumatic dermatome was used in 89 cases, while traditional sharp excision and/or hydrosurgery was performed in 121.

• Sites and area excised: The dermatome group demonstrated a significantly higher number of sites excised (median 3 vs. 2, *p*=.009) and larger total wound area excised (median 1694 cm^2^ vs. 894 cm^2^, *p*=.002).

• Operative times: No statistically significant differences in operative times were observed across wound-size strata.

• Transfusions: Transfusion rates were not significantly different between groups across wound-size strata.

**Conclusions:**

Use of the circular pneumatic dermatome was associated with significantly larger wound surface area excised per procedure, likely reflecting reduced surgeon fatigue and enhanced efficiency. While operative times and transfusion rates were not statistically different, the dermatome facilitated more extensive excision within a single surgery. Future studies should evaluate objective blood loss and long-term patient outcomes.

**Applicability of Research to Practice:**

Incorporation of a circular pneumatic dermatome into burn wound excision protocols may improve procedural efficiency and reduce surgeon fatigue.

**Funding for the study:**

N/A.

